# Knowledge, Behavior, and Influencing Factors of Coarse Grain Consumption among Chinese Adults: A Focus Group Study in Xi’an

**DOI:** 10.1016/j.cdnut.2024.104474

**Published:** 2024-10-12

**Authors:** Jiawen Xie, Junqi Li, Guoqing Ma, Menghan Wang, Yunfeng Li, Yafang He, Kun Xu, Tian Tian, Nan Yang, Qian Wang, Jie Chang, Xin Liu

**Affiliations:** 1 Key Laboratory for Disease Prevention and Control and Health Promotion of Shaanxi Province, Key Laboratory of Environment and Genes Related to Diseases (Xi'an Jiaotong University), Ministry of Education, School of Public Health, Global Health Institute, Health Science Center, Xi'an Jiaotong University, Xi'an, China; 2 The Second Affiliated Hospital of Xi'an Jiaotong University, Xi'an, China; 3 SpecAlly Life Technology Co., Ltd., Wuhan, China; 4 Department of Nutrition, Xi’an Daxing Hospital, Xi'an, China; 5 Health Management Department, the Second Affiliated Hospital, Xi'an Jiaotong University, Xi'an, China; 6 Department of Pharmacy Administration and Clinical Pharmacy, School of Pharmacy, Health Science Center, Xi'an, China

**Keywords:** coarse grain, food perception, food choices, focus group, qualitative research

## Abstract

**Background:**

Coarse grains are rich in fiber, minerals, and other beneficial nutrients but are consumed at low levels in modern populations. The factors that influence coarse grain consumption in current living and dietary environments are not fully understood.

**Objectives:**

This study aimed to explore the knowledge and behavior related to coarse grain consumption and identify the influencing factors among Chinese citizens.

**Methods:**

Six focus group discussions were conducted with 39 participants aged 18–65 years from diverse social backgrounds in Xi'an, China. All discussions were transcribed verbatim and analyzed using inductive thematic analysis.

**Results:**

The majority of participants demonstrated insufficient knowledge about coarse grains, including their definitions, health benefits, and recommended intake. A small number of the participants reported regular consumption. The barriers to coarse grain consumption were poor sensory properties, insufficient cooking skills and time, limited availability of ready-to-eat foods, established dietary habits, and high prices. Additionally, new barriers included psychological burden, concerns about food safety, the impact of processing methods on health benefits, and special health conditions. Health benefits and family influence emerged as the 2 primary factors motivating coarse grain consumption. Most participants expressed a positive attitude toward partially replacing staple foods with coarse grains. Enhancing health education, innovating food processing methods, improving labeling systems, and strengthening safety supervision have been recommended for increasing coarse grain consumption.

**Conclusions:**

A gap exists between health awareness and healthy behaviors regarding coarse grain consumption; thus, collaborative efforts among government agencies, educational institutions, nutrition societies, the food industry, policymakers, and health professionals are essential to overcome these challenges.

## Introduction

With rapid urbanization and lifestyle changes, the global burden of noncommunicable diseases has significantly increased, including in China [1]. An unhealthy diet plays a major role in the development of these diseases [1,2], with insufficient whole grain consumption being a significant concern owing to its proven benefits in reducing the incidence of cardiovascular events [3]. In the macronutrient consumption guidelines, whole grains and pulses are listed as the primary sources of carbohydrates and are considered important for increasing dietary fiber intake [4].

Since the 1940s, the Green Revolution has increased the yield, availability, and affordability of refined grains, whereas whole grain consumption has declined in many countries [5]. According to the Global Dietary Database, the global consumption of refined grain was 336.6 g/d, whereas that of whole grain was 48.8 g/d in 2018 [6]. The 2022 Chinese Dietary Guidelines recommended a whole grain intake of 50–150 g/d [7]. Nevertheless, whole grain consumption remained significantly below the recommended levels worldwide. In China, the China Health and Nutrition Survey data indicated that the daily intake of whole grains was only 20.1 g in 2018 [8]. This highlights an urgent need to identify the perceptions and barriers toward whole grain consumption. Previous studies in Western countries perceived cost differences, dietary habits, inadequate knowledge, and poor organoleptic properties as barriers [[9], [10], [11], [12]]. However, limited evidence is available in Asian countries, including China, where dietary habits, food variability, and cooking styles differ from those in Western populations [13]. Additionally, no official legislation regulates whole grain foods, except for an association standard that mandates a minimum of 51% whole grain ingredients per total weight of product [7].

In fact, the concept of whole grains is new in Chinese dietary culture. Traditionally, a similar concept known as “coarse grains” has been prevalent. Coarse grains typically refer to grain crops other than wheat and rice, such as sorghum, buckwheat, oats, barley, corn, and dried beans (such as red beans and mung beans) [14]. Dried beans are not included in the standard categories of whole grains. Both whole grains and coarse grains contain endosperm, germ, and bran [15,16], and they offer similar nutritional benefits, including high levels of dietary ﬁber and plant polyphenols. Dried beans are also recognized as a good source of protein [17]. These nutrients contribute to the regulation of glucose levels, blood lipids, blood pressure, and oxidative stress [16]. A large cohort study involving 500,000 Chinese participants found that a higher consumption of coarse grains was associated with lower risks of hypertension, type 2 diabetes, and ischemic stroke [18,19].

Previous studies have predominantly examined the bioactivity of coarse grains and the effects of coarse grain consumption on disease risk [[18], [19], [20], [21], [22]]. However, the factors influencing coarse grain consumption in current living and dietary environments among Chinese adults remain insufficiently understood. Little is known about the perceptions of and behavioral barriers to coarse grain consumption in this population.

In the present focus group study, we investigated the insight factors influencing the consumption behavior of coarse grain in Chinese adults living in Xi’an, with a particular interest in identifying barriers and facilitators to promoting a long-term increase in coarse grain consumption among Chinese adults.

## Methods

### Study design and participants

This focus group study aimed to identify the perceptions and barriers to coarse grain consumption in Chinese adults. Xi’an is the capital city of Shaanxi Province, the largest city in northwestern China, and 1 of the 10 National Central Cities in China, with a population exceeding 10 million people in 2020 [23]. The local people in Xi’an typically prefer wheat products over rice as their staple food [24]. Residents of Xi’an aged between 18 and 65 y, with no difficulties in verbal communication, were included in the study. Convenience sampling was employed to recruit participants. University researchers posted WeChat (a popular Chinese social media platform) advertisements, and some of our collaborators in hospitals reposted the WeChat advertisements to reach a broader audience. Interested participants were instructed to scan the quick response code to complete an online sign-up form. The researchers contacted the eligible volunteers to make appointments for onsite focus group discussions. Participant recruitment was concluded when no new themes emerged from the analysis.

At the time of enrollment, the participants were provided with detailed information about the study. Meanwhile, they were required to complete a brief questionnaire that contained questions related to age, sex, employment status, educational attainment, income, and staple food preferences. This study was approved by the Ethics Committee of Xi’an Jiaotong University Health Science Center and conducted in accordance with the Declaration of Helsinki. All participants signed an informed consent form prior to their participation.

### Procedure

**FIGURE 1:**
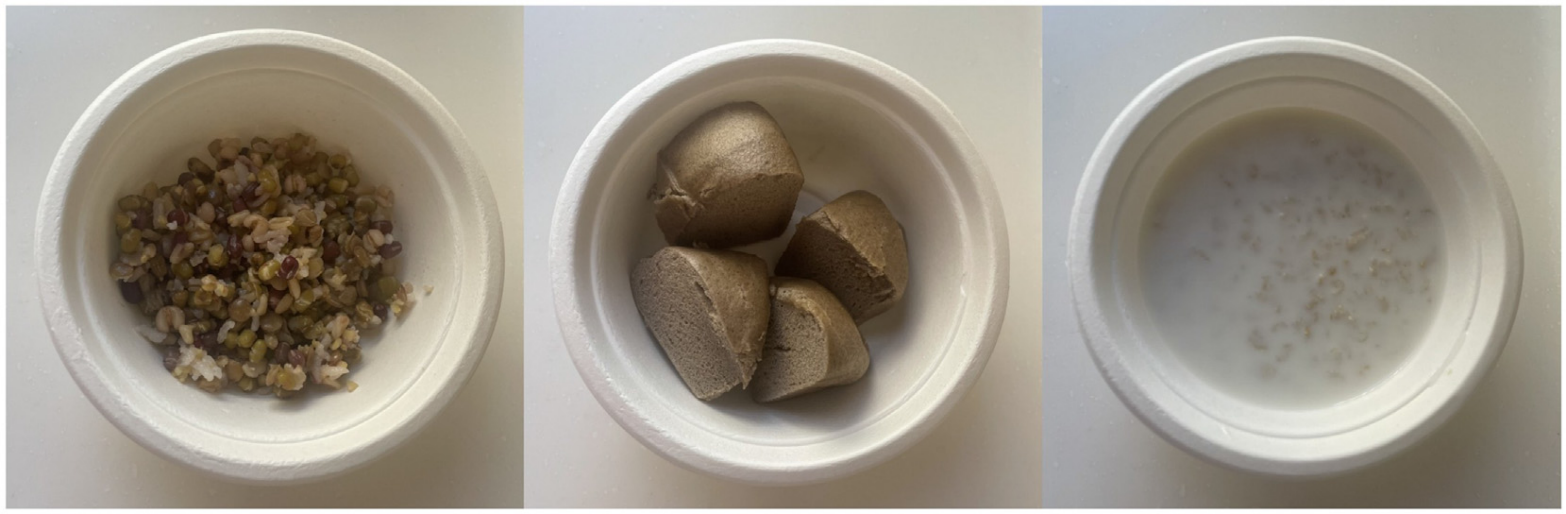
Photos of coarse grain food served before the focus group discussion. Coarse grain rice (left), steamed buns (middle), and instant oats with milk (right).

The focus group discussions were conducted between May and December 2022. All sessions were held in a private and comfortable meeting room at prescheduled time. To minimize authority bias, participants with similar characteristics (such as their affiliations with universities, hospitals, or social communities or occupations such as teachers, students, or others) were grouped in the same focus group. Each focus group discussion was moderated by the same trained researcher specializing in public health, with an assistant monitoring the proceedings and taking notes. Ten minutes before each discussion, the participants were provided with 3 types of readily available coarse grain food items (Figure 1): coarse grain rice (a mixture of refined rice boiled together with various coarse grains: millets, sorghums, oats, black rice, pearl barley, red beans, mung beans, red kidney beans, and black turtle beans), steamed buns (a blend of coarse grain flour and refined wheat flour, fermented and steamed), and instant oats with milk. These items were selected due to their common use in the Chinese diet and their ease of preparation in research settings. Following tasting, the participants evaluated the palatability of these items using a predetermined 5-point Likert scale (1=very dissatisfied to 5=very satisfied) in 3 aspects: tasting, texture, and flavor. The overall score was calculated as the sum of the 3 scores.

After a brief introduction to the aims and participation guidelines of the focus group discussion, the moderator facilitated the discussion in a relaxed and congenial atmosphere. The participants were asked 11 semistructured questions in each discussion to guide the discussion and ensure comprehensive coverage of research topics (Table 1). These questions were developed based on an internal discussion and review of similar studies [12,25], with a focus on the following 4 aspects: *1*) knowledge of coarse grains, *2*) coarse grain consumption habits, *3*) coarse grain consumption preferences, and *4*) factors affecting the consumption of coarse grains. At the end of the discussion, the participants were asked about their willingness to substitute one-third to one-half of the staple food with coarse grains. The entire discussion duration, other than the time spent tasting food, lasted for 40–60 min and was audio-recorded with the participant’s consent.

**TABLE 1:** The semistructured discussion questions.

1) Knowledge of coarse grains	What do you think is coarse grains? Name the coarse grains you know.
	Do you know the benefits of coarse grains?
	Do you know the drawbacks of coarse grains?
	What channels did you obtain information about coarse grains from?
2) Coarse grain consumption habits	Do you eat coarse grains in your daily life? How often do you eat them?
	What is the reason for the frequent consumption of coarse grains? (For people who do not eat often, what are the reasons for not eating?)
3) Coarse grain consumption preference	Do you think any forms of coarse grain foods are suitable for your taste?
	What characteristics do you expect/dislike more from coarse grain foods? (Taste, texture, flavor)
4) Factors affecting the consumption of coarse grains	Regarding your daily dining habits, what are the factors that affect your consumption of coarse grains?
	Would you choose to increase your purchase of coarse grains due to their health benefits?
	Can you accept replacing 1/3–1/2 of the staple food with coarse grains? If possible, what types of coarse grains and food forms are they?

### Data analysis

The recorded data from the focus group discussion were transcribed verbatim by the first author within 2–3 d after each session. The analysis was conducted using NVivo 12 (QSR International Pty), a qualitative data management software. Inductive thematic analysis was used to analyze the transcripts, following the 6 phases defined by Braun and Clarke [26,27]. Initially, researchers Jiawen Xie and Menghan Wang, both specializing in public health, independently identified the broad codes related to coarse grain consumption from the transcripts based on their interpretive perspectives. Subsequently, the codes were iteratively reviewed by both authors and collected to explore potential themes and subthemes. Finally, an associate professor experienced in qualitative studies (Xin Liu) reviewed the topics and codes to ensure that an accurate representation of the participants’ overall perspectives was established. Disagreements among the researchers were discussed and resolved through consensus. To maintain anonymity, the participants’ identifying information was removed and labeled using new codes such as FG1M1 (focus group No.1 male subject No.1) and FG1F1 (focus group No.1 female subject No.1).

For quantitative analysis, the participant characteristics were expressed as the mean ± SD for continuous variables and as numbers (%) for categorical variables. Participants’ preferences for different foods were compared using analysis of variance. The fixed variables included types of coarse grains, whereas the responses were the scores for different dimensions. Quantitative analysis was performed using the R software (version 4.2.1).

## Results

The results were presented as participant characteristics, coarse grain preferences, and the 6 major themes identified from the analysis. These themes were as follows: *1*) knowledge and understanding of coarse grains, *2*) coarse grain consumption behavior, *3*) barriers to coarse grain consumption, *4*) motivations for increasing coarse grain consumption, *5*) attitudes toward replacing some staple foods with coarse grains, and *6*) strategies for increasing coarse grain consumption. To maintain conciseness, we only provided the representative discussion details for each theme in the text.

### Participant characteristics and their preference for tasting coarse grain food

**FIGURE 2:**
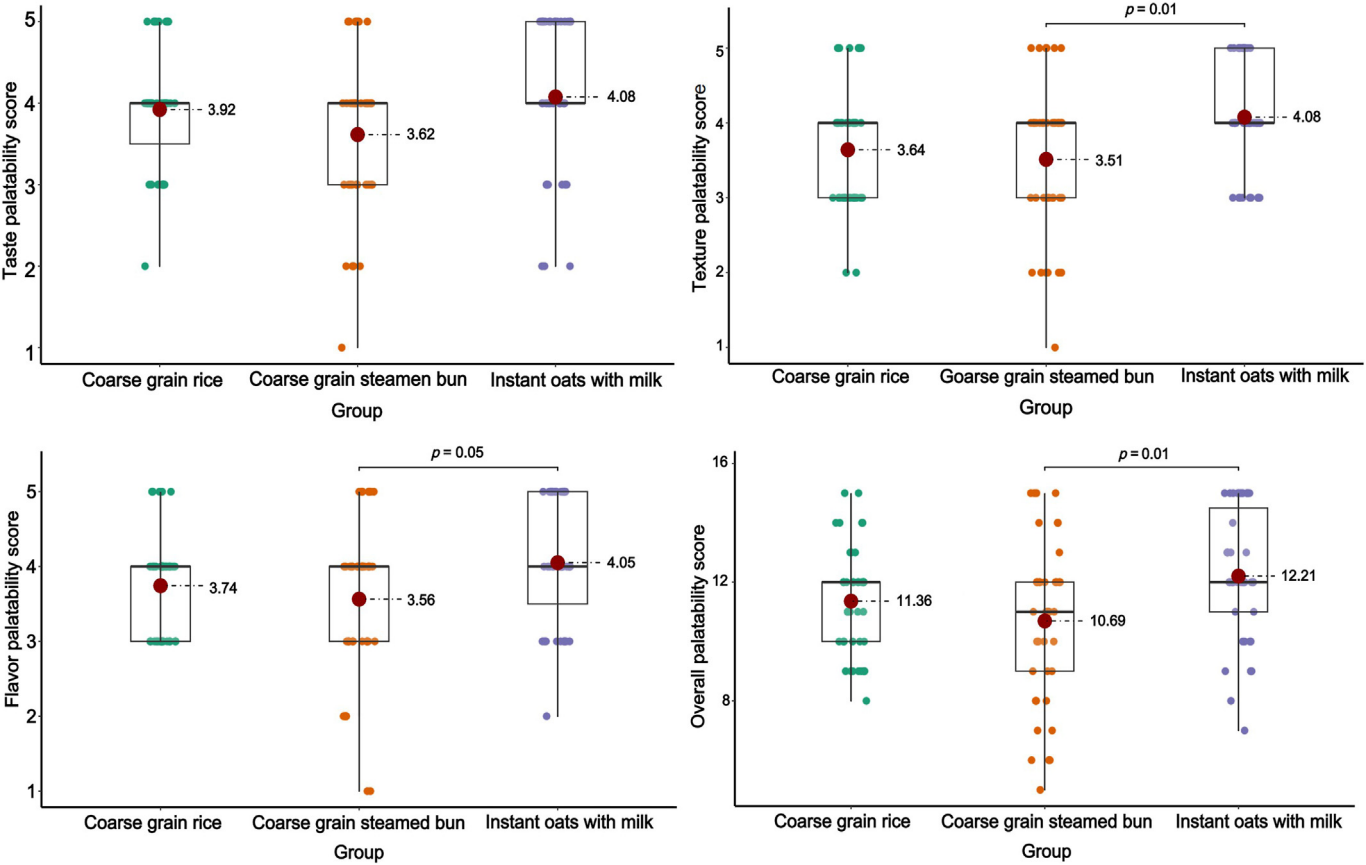
Palatability scores for the given coarse grain foods.

The 6 focus groups were made up of people with different levels of social-economic status, health conditions, and educational attainment. They included university biomedical students, university nonbiomedical students, university professionals, university cafeteria staffs, inpatients with hypertension, and general community residents. In total, 39 participants were included, with each group comprising 5–8 individuals (Table 2). The demographics of the participants are presented in Table 3. The mean age of the participants was 35.69 ± 13.47 y, and 66.67% were female. Two-thirds of the participants (66.67%) completed a bachelor’s degree or higher, and approximately one-third reported a monthly family income of 10,000 CNY (∼166.05 USD) or higher. Rice and noodles were the preferred staple foods. Overall, the participants favored instant oats with milk the most. In terms of texture and flavor, instant oats with milk scored significantly higher compared with coarse grain steamed buns; however, the difference in taste was not significant (Figure 2).

**TABLE 2:** Overview of focus groups.

Focus group	Number	Age range (y)	Sex
University biomedical students	8	22–25	7 females, 1 male
University nonbiomedical students	8	23–28	5 females, 3 males
University professionals	5	33–42	2 females, 3 males
University catering staffs	6	32–58	4 females, 2 males
Inpatients with hypertension	5	43–62	3 females, 2 males
Community residents	7	19–52	5 females, 2 males

**TABLE 3:** Basic demographic information of the participants.

Characteristics	Mean ± SD or n(%)
N	39
Age (y)	35.69 ± 13.47
Gender (%)	
Female	26 (66.67)
Male	13 (33.33)
Body mass index (kg/m^2^)	22.49 ± 3.62
Education level (%)	
Middle school diploma	7 (17.95)
High school diploma	6 (15.38)
Bachelor's degree	20 (51.28)
Master's degree	6 (15.38)
Monthly household income (%)	
<5,000 yuan/mo	11 (28.21)
5,000–10,000 yuan/mo	15 (38.46)
>10,000 yuan/mo	13 (33.33)
Staple food preference (%)	
Rice	30 (76.92)
Steamed bun	20 (51.28)
Noodles	29 (74.36)
Tubers	6 (15.38)
Others	1 (2.56)

### Knowledge and understanding of coarse grains

All 6 focus groups discussed what coarse grains were and their health benefits. Most of the participants were familiar with 2 or 3 common types of coarse grains, such as sorghum, oats, buckwheat, mung beans, red beans, and soybeans. However, confusion existed regarding the precise definition of coarse grains. Some participants perceived coarse grains as grains other than rice and wheat flour but struggled to provide more detailed definitions. Additionally, confusion arose regarding the difference between coarse grains and starchy vegetables, such as sweet potato and Shan Yao (Chinese yams).

Participants generally agreed that coarse grains are healthy due to their high fiber and vitamin content. They also recognized that coarse grains may contribute to the regulation of blood glucose, blood pressure, blood lipids, and weight and help maintain satiety. Some participants struggled to explain the underlying scientific principles and were unable to provide indepth explanations.

“It (coarse grain) is related to cellulose with higher content, which can reduce blood lipids and flush fat out of the body?”——FG5M1

“It (coarse grain) may lower glycemic response rates. A slow glycemic rate is also beneficial for weight loss.”——FG1F1

“The best thing to eat for diabetes is this coarse grain.”——FG5F2

“I think it (coarse grain) contains more fiber, so your body needs more time and energy to digest it. At this moment, you feel very full.”——FG1F6

Recognition of the health benefits of coarse grains was typically derived from knowledge acquired from the Internet, family, and friends. In addition, some participants’ understanding of coarse grains was heavily influenced by their traditional Chinese medicine beliefs. Specifically, they mentioned that consuming red beans and Chinese pearl barley watery porridge could help clear dampness (a Chinese medicine term), mung bean soup may reduce internal heat (a Chinese medicine term) in summer, and black beans may stimulate hair growth. However, most participants were not entirely certain about these theories and expressed the need to obtain more scientific evidences.

“Drinking mung bean soup in the summer can clear internal heat.”——FG4M2

“Eating red beans and Chinese pearl barley watery porridge can help remove damp.”——FG1F2

“Also, is it true that eating certain foods provides specific benefits—such as black beans, black rice, and black sesame, which are said to promote black hair growth, or red foods, which are believed to nourish the blood? I am curious whether there is scientific evidence behind these claims.”——FG2F1

During the focus group discussion, contradictory perceptions regarding coarse grain foods emerged. Some participants believed that coarse grains, especially millet porridge, could provide nourishment, whereas others believed that coarse grains rich in dietary fiber were difficult to digest. Meanwhile, mixed opinions were expressed on whether milled coarse grains and products that contained limited coarse grain proportions, such as coarse grain flour, bread, and crackers, should be considered coarse grains.

In addition, almost all participants were unaware of the recommended daily intake level of coarse grains for the Chinese population. Some participants were asked which coarse grain was the best and which had higher health benefits.

“Millet porridge can be used to nourish the stomach.” ——FG4F3

“It (coarse grain) can promote intestinal peristalsis.” ——FG2F8

“We are probably used to eating white rice since childhood. If I suddenly eat coarse grains every day, I think my digestive system will struggle; it can be unbearable.” ——FG2M3

“Is whole wheat bread or whole grain bread also considered as coarse grain?” ——FG2M3

### Coarse grain consumption behavior

The majority of participants reported having consumed coarse grains before, but only a minority reported regular consumption, such as daily. Others, including patients with hypertension, reported occasional intake (once or twice a month).

The most frequently mentioned formats of coarse grains were coarse grain porridges and instant oats. Other forms included Fa Gao (Steamed Chinese sponge cake), bean buns, coarse grain pancakes, and corn cake. Interestingly, participants reported a tendency to eat coarse grains more frequently at breakfast and dinner than at lunch, whereas the vast majority of participants rarely consumed coarse grains when dining in restaurants.

“In winter here, we eat porridge made from corn grits, whereas other types of coarse grain are rarely consumed.”——FG6F3

“I eat ready-to-eat oat cereals mixed with milk. I have it for breakfast.”——FG2F1

“That’s all occasional; we only eat it (coarse grain) once or twice a week.”——FG5F2

“If I eat out, I rarely get to eat coarse grain”——FG3F4

### Barriers to coarse grain consumption

**FIGURE 3:**
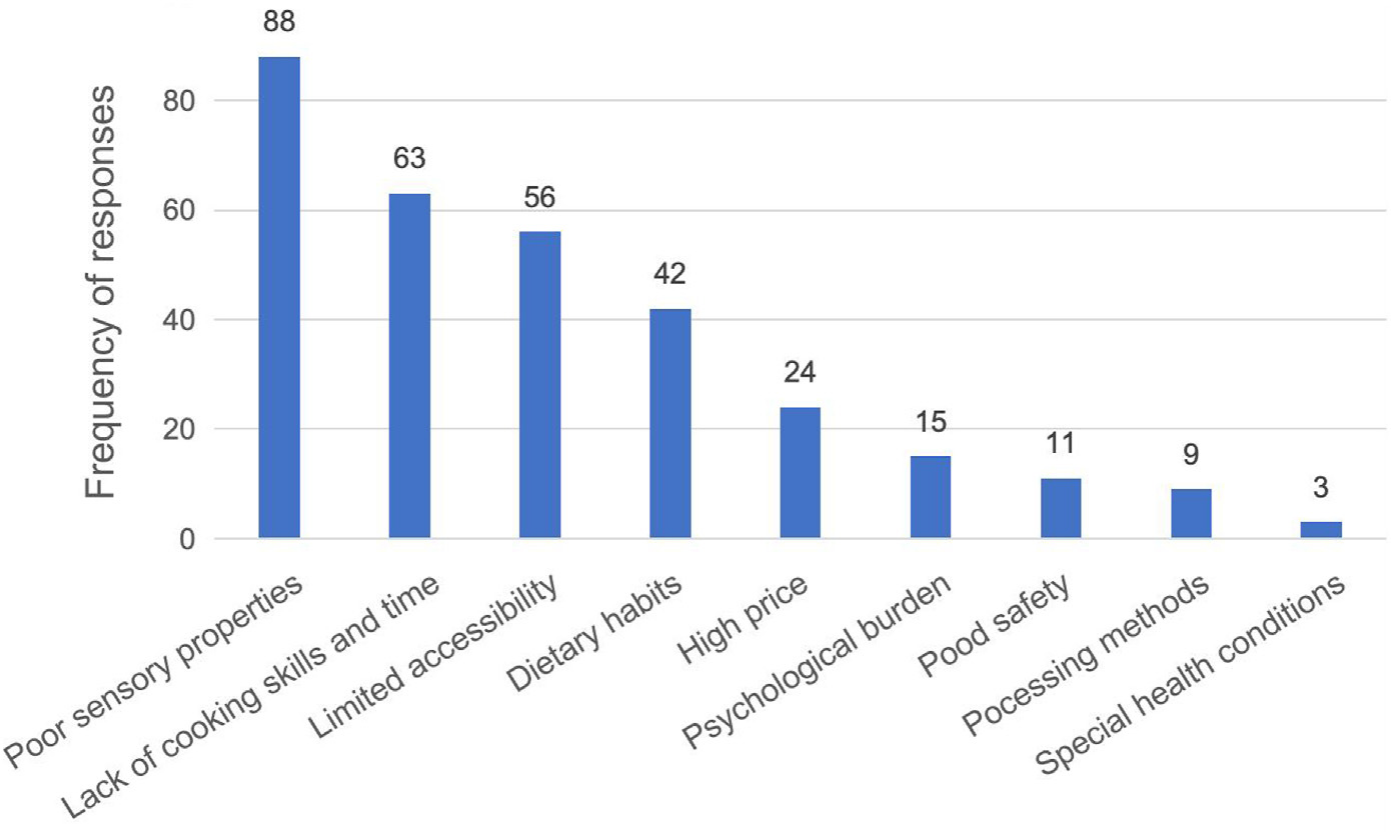
Frequencies of perceived barriers toward coarse grain consumption.

The identified barriers to coarse grain consumption were poor sensory properties, the insufficient cooking skills and time, limited availability of ready-to-eat foods, and established dietary habits. In addition, high prices, psychological burden, food safety, the impact of processing methods on health benefits, and individual health differences may also limit the consumption of coarse grains (Figure 3).

When discussing the factors influencing their consumption of coarse grains, many participants identified sensory properties (i.e., taste, texture, appearance, and smell) as the most important factors, with the predominant barrier being rough texture. Some participants used throat piercing to describe the grainy texture of coarse grains.

“The steamed corn bread we just tasted, I used to eat a lot of it when I was a kid. The ones I had before often made my throat scratch! Gosh, it felt like my esophagus was scraped when I swallowed it.”——FG2M4

“It was rough, not fine enough, and I couldn’t swallow it.”——FG6F2

Additionally, 3 participants stated that they strongly disliked the smell of beans, which prevented them from eating any food containing beans.

“It’s about the taste, and there is an undesirable odor’ some people can’t adapt to the beany flavor.”——FG5M4

During the discussion, the lack of cooking skills and time emerged as major factors. Participants reported that preparing coarse grains was more complex and time-consuming than cooking rice and wheat flour. For participants who did not cook themselves, health was not a major determinant in choosing food. Instead, convenience was prioritized.

“For me, it is troublesome. Soaking and cooking it is a hassle, which is why I stick to refined white rice for my meals, even though I know the benefits of coarse grains.”——FG3M2

“Another thing is that everyone is eating rice and flour, and people have come up with new ways to eat them, using simple ingredients to create various styles of dishes. But with coarse grains, when you make them yourself, it feels like there’s not much innovation that attracts you to cook coarse grains.”——FG1F4

“You might wonder why I often eat coarse grains, well, because my mom cooks them.”——FG3F3

“Why would I choose to buy precooked coarse grain foods online; that is, I can eat it immediately, the ones that are quick and easy to prepare. It only needs to be heated; so, the preparation time of the coarse grains is a significant factor.”——FG1M3

Poor accessibility was frequently mentioned as a barrier to consuming coarse grain foods. A few participants stated that purchasing coarse grains is relatively easy; however, buying processed and coarse grain products from restaurants and canteens is challenging. Moreover, these options are almost nonexistent.

“The key problem is that there are no such things in schools. If it is available in school, it will definitely become popular.”——FG2F1

“We can seldom buy one (coarse grain) when eating out.”——FG6F2

Participants also noted that the concept of coarse grains is frequently marketed in many food products. However, these products typically contain only a small amount of actual coarse grains, with excessive sugar and oil added to enhance the taste and flavor, potentially diminishing their health benefits. The quality of so-called coarse grain products in the market is questionable.

“I don’t think it’s reliable. I think most of the coarse grain products you find in the market are made with refined wheat flour, with only a small amount of coarse grains added for color or flavoring. We cannot taste the original and authentic flavors of pure coarse grains.”——FG2F1

“If they (coarse grains) do not taste good, they will not be eaten. Honestly speaking, something tastes good; it has many ingredients and additives.”——FG2F6

Dietary habits are among the most common barriers to coarse grain consumption. A few participants stated that they had not intentionally purchased coarse grains. Although they were aware of their health benefits, they were reluctant to incorporate them into their diets.

“It’s like I just don’t have the consciousness to eat coarse grains. Sometimes, even if the coarse grain has been stored for a long time and gone bad, I do not even want to cook it.”——FG6F2

“Many times, our eating habits are casual, not about eating more coarse grains on a specific day; it’s all random, without a systematic approach.”——FG5M1

Attitudes toward the price of coarse grains are ambivalent. Participants consistently noted that the price should not be a barrier to coarse grain consumption. However, they complained that the significant increase in the price of processed coarse grains was highly related to marketing channels and serving formats.

“Nowadays, the prices of coarse grains are acceptable by the public, and they are considered beneficial for one’s health.”——FG4M2

“That (coarse grain) is so expensive. For example, the price of a steamed bun is 0.5 yuan, whereas a steamed corn bun costs 1.3 yuan.”——FG4M4

“Processed coarse grain foods are available but tend to be expensive. For example, prices at convenience stores differ from those at regular supermarkets. If you go to a place such as Holiland or other cake shops, it seems that the price immediately increases once coarse grains are included.”——FG3M2

During the discussion among students, some expressed that consuming coarse grains or focusing on health can cause psychological pressure.

“It is too troublesome. Even when eating it (coarse grain), I feel exhausted because I have to sacrifice my precious eating time to consume something that does not taste good for the sake of health after working all day. I feel like I have been very utilitarian all day, striving for many things; later on, I do not want to eat it anymore.”—— FG2F2

“If I choose to eat coarse grains, I instinctively feel like it is for the sake of health; so, I should not go for any oily food.”——FG1F6

University teachers demonstrated an advanced, rational understanding of the health benefits of coarse grains. In addition to the aforementioned barriers, concerns have been raised about the safety risks in the production of coarse grains (especially the impact of environmental pollution). They questioned whether it is necessary to commercialize coarse grains because those who are genuinely health-conscious should remain vigilant about the potential risks associated with processed products.

“Will there be more contamination in coarse grains? I remember seeing many farmers drying their crops on the road.”——FG3F3

“There is a misconception that coarse grains are always green and healthy, but in reality, because they are not primary staple foods, I think the national standards and regulations for them are lagging behind those for staple grains.”——FG3M2

In addition, the impact of the processing methods on the health benefits of coarse grains is discussed in detail. Some special health problems may limit coarse grain consumption.

“Does the cooking method of this food (coarse grain) have a significant impact on its nutritional absorption?”——FG2M3

“You can see that my family cooks coarse grain porridge every day. My concern is that although coarse grains can lower blood glucose, the final effect may be uncertain when they are prepared as porridge.”——FG3F3

“I used to have high uric acid. During the physical examination, my doctor advised me to reduce my bean consumption. So, I intentionally reduced it, and my uric acid levels have since normalized.”——FG3M5

### Motivation to increase coarse grain consumption

When discussing the reasons for increasing their coarse grain consumption, the primary motivation of the vast majority, especially patients with hypertension, is to improve health. Good household wealth is believed to increase coarse grain consumption.

“As I’m quite concerned about colorectal cancer. I hope to increase my dietary fiber consumption. Of course, I hope to reduce the risk of having that disease by increasing my intake of coarse grain. In addition, it can be beneficial for managing blood lipids and sugars.”——FG3F3

“Do not solely focus on food consumption; you have to consume them in a scientific and healthy manner.”——FG5M4

“For example, if you have enough money to hire nannies, you would likely increase your consumption of coarse grains, as you would have someone specialized in preparing these foods for you. When conditions allow, everyone naturally prefers to remain healthy.”——FG4M2

Many participants reported that dining with family members who have a high intake of coarse grains can significantly increase their consumption.

“I think I eat a lot of coarse grain because I consume porridge more often. It is not my professional knowledge that leads me to eat more; rather, it is a family habit.”——FG3M4

“Although I do not particularly enjoy porridge, I do not mind having it when dining with my family.”——FG3M5

Interestingly, the sensory properties of coarse grains can be a barrier for some people but a benefit for others. A few participants claimed that they enjoyed the chewy and grainy texture of coarse grains.

“If I have several options available to me, like porridge, noodles, steamed buns, rice, and coarse grain rice, I would choose coarse grain rice. This preference is partly owing to my southern regional background, where I developed a taste for food with a grainy texture.”——FG1M3

“Personally, I have a strong preference for coarse grains likely due to my northern regional background and my appreciation for their glutenous and chewy texture. It is simply a matter of preference as I generally favor a lighter diet and tend to prefer slightly rougher foods.”——FG1F5

The expectation of weight loss is a major reason why young people consume coarse grains.

“Actually, I do consider consuming coarse grains when I want to lose weight. Sometimes, I would consume ready-to-eat oats at night.”——FG2F7

“When I am trying to lose weight, I would look for coarse grain bread or partially prepared coarse grain rice.”——FG2F8

### Attitude toward replacing some staple food with coarse grains

The vast majority of participants expressed willingness to increase their consumption of coarse grains owing to their health benefits. Biomedical students revealed a stronger inclination to improve their health.

“I definitely want to adjust my diet for the sake of health.”——FG5F5

However, the level of health awareness among community residents was lower than that of other groups (university students, teachers, and patients with hypertension). Community residents prioritized more appealing tastes and generally had negative attitudes toward using coarse grains instead of refined rice and flour. A few young people acknowledge the health benefits of coarse grains, but the long-term consumption of coarse grains for health reasons can place significant pressure on them (refer to the psychological burden mentioned in Section **Barriers to coarse grain consumption**).

“I cannot. I find that eating coarse grains reduces my happiness. It may not provide the same level of satisfaction as consuming simple carbohydrates.”——FG1F2

“I may consider its price and my time. Compared with my work, are the health benefits truly a top priority? In practical situations, people may want to prioritize health, but other constraints may make it unaffordable.”——FG4M2

Although most participants had a positive attitude toward replacing refined rice and wheat flour with coarse grains, when asked if they were willing to substitute one-third to one-half of their staple food with coarse grains, most participants felt that one-half was excessive and one-third was more probable to persist.

### Strategies for increasing the consumption of coarse grain

Most participants actively proposed several suggestions to increase coarse grain consumption among populations: *1*) enhance the popularization of nutritional science of coarse grains to improve public understanding of their health benefits; *2*) innovate the format and improve the processing technology of coarse grains, enhance the taste of coarse grains although retaining their nutritional components, integrate coarse grain products into people’s daily diet (such as beverage, tea, milk tea, and Liangpi [a popular cold noodle]); *3*) establish and improve a labeling system for healthy coarse grain products to reduce consumers’ learning costs; and *4*) strengthen the safety supervision of coarse grains from cultivation to production and processing.

“We need to promote this (coarse grain) through education. We need everyone to improve their understanding of coarse grains and accept them.”——FG4M4

“If the processing is well-executed, and the processing methods are diversified, the coarse grains can become more appealing.”——FG5M1

“The main goal is to make the coarse grain more palatable and similar to commonly consumed foods, to make them more enjoyable and convenient to eat.”——FG1F2

“However, I believe consumers should not be responsible for bearing the cost of learning to identify genuine coarse grain products.”——FG3M2

“Regardless of the brand of rice I choose, it is still rice. However, when purchasing coarse grain products, the coarse grain content may vary, with one product containing 80%, whereas others containing only 60%. A system that can quickly determine the content of coarse grains (in products) is required.”——FG1M6

“So, before the coarse grain can be widely popularized, we must develop a suitable technology and establish regulatory supervision to support its widespread adoption.”——FG3M2

## Discussion

In this focus group study of Chinese adults in Xi’an, participants exhibited a limited understanding of the definition and health benefits of coarse grains, with only a small proportion reporting regular consumption. The common barriers to increasing coarse grain intake included poor sensory properties, insufficient cooking skills and time, limited availability of ready-to-eat coarse grain foods, and existing dietary habits. These findings align with those of previous studies on whole grains [11,12,[28], [29], [30]]. Additionally, we identified other barriers, including high cost, psychological burden, food safety concerns, the impact of processing methods on health benefits, and special health conditions. Health benefits and positive family influences were noted as the 2 major factors facilitating coarse grain consumption. Enhancing health education, innovating food processing methods, improving labeling systems, and strengthening safety supervision promote greater consumption of coarse grains in the population.

Our findings on knowledge and behavior are consistent with those of previous studies on whole grains. The general public has limited knowledge of coarse grains and whole grains, including their definition, recommended daily intake, specific nutritional benefits, and methods for obtaining them [12,30]. Most participants reported occasional consumption of coarse grains. During the discussion, participants reported difficulties in accessing health information and guidance on coarse grains, especially among older adults and low-income groups. Education is essential to improve public understanding of the nutritional value of coarse grains [31]. This can be achieved by providing information on their health benefits and demonstrating how to incorporate them into various meals [32]. Practical and effective educational methods, such as short videos, live broadcasts, and school-based programs, are recommended to enhance understanding of the health benefits and usage of coarse grains [33]. Meanwhile, health education should focus on improving accessibility, providing targeted interventions, and ensuring that all individuals have access to the necessary support and guidance [34].

Although sensory properties are a major factor limiting the consumption of coarse grains, most participants stated that they found the coarse grain food we provided to be acceptable and reported that the taste was not as unpleasant as they had anticipated. This was also observed in palatability scores. A previous focus group study from China examining brown rice revealed that participants initially perceived brown rice as inferior in both taste and quality prior to actually tasting it; however, after tasting and learning about its nutritional value, the participants expressed a greater willingness to consume brown rice [28]. Thus, overcoming negative preconceived notions about the taste of coarse grains may be crucial for increasing their consumption. Exposure can shape taste preferences [20]. These data suggest that increasing the accessibility and availability of coarse grain foods may provide more opportunities for people to develop a positive perception of their taste, texture, and flavor.

Price has been recognized as one of the most significant factors influencing whole grain consumption [12,[28], [29], [30]]. However, the present study revealed a more nuanced perspective on cost. Although the cost of raw coarse grains did not hinder their consumption, the participants expressed concerns about the higher prices of processed coarse grain products. Economic interests are crucial for food enterprises [35], but government agencies may need to take responsibility for balancing food product distribution and positioning by considering the diversity of population affordability and nutrition requirements. Developing cost-effective coarse grain products with good sensory properties, minimal added sugar and oil, and healthier ingredients is both critical and urgent.

Interestingly, we identified psychological factors as barriers to the consumption of coarse grains. One participant stated, “If I choose coarse grains, I have to avoid eating greasy foods.” However, for some people, the pursuit of good health is perceived as an unnecessary burden. Previous studies have demonstrated that adopting healthy dietary behaviors can increase the psychological burden due to the heightened self-discipline required in other dietary areas [36]. The WHO defined health as “a state of complete physical, mental, and social well-being and not merely the absence of disease or infirmity,” a definition established as early as 1948 [37]. We may need to avoid turning the intention of a healthy diet into an unhealthy obsession [38] since psychological and physiological health are essential. Furthermore, governments should commit themselves to creating a social and physical environment that makes choosing coarse grains and healthy foods easy and natural [39].

With regard to the willingness to partially replace staple foods with coarse grains, most participants responded positively but indicated a need for practical guidance. Interestingly, the dining environment and occasion appear to significantly influence coarse grain consumption. To increase accessibility, nutrition initiatives and promotions should emphasize the inclusion of coarse grain as a staple food option on the menus. Additionally, family nutrition education should be strengthened, especially for those who implement family nutrition plans and directly determine the diet of all family members. Parental food choices play a crucial role in shaping children’s dietary habits [40]. Educating parents about the health benefits of coarse grains and food selection can facilitate the repeated exposure of children to healthy foods in various physical and social settings [41,42].

To our knowledge, this qualitative study is the first to explore coarse grain consumption in China. However, this study has some limitations. Recruitment bias may have occurred owing to voluntary participation. Individuals who were more concerned about nutrition, health, and food may have been more likely to participate in the study. Convenience sampling likely resulted in a predominantly well-educated group of consumer participants. Moreover, as information on coarse grain health benefits was provided to participants during the discussion, they were likely to report more socially desirable answers. Finally, given the qualitative nature of the study, the results of this research should not be considered representative but rather exploratory and descriptive. This study was carried out in Xi’an, and cannot represent the population in China. However, the number of university students in Xi’an is expected to reach one million in 2023, placing the city seventh among all cities in China [43]. These results may offer valuable insights for understanding situations in other cities with similarly advanced educational development. The total sample size is limited. Although data saturation can be observed when no new themes emerge from the last focus discussion, conclusions should be applied with caution when generalizing to the broader population or other cultural groups.

In conclusion, despite their well-recognized health benefits, coarse grains face low consumption due to poor sensory properties, insufficient cooking skills and time, limited availability of ready-to-eat foods, and established dietary habits. Increasing the consumption of coarse grains is a complex social challenge that requires collaborative efforts by government agencies, the education sector, nutrition societies, the food industry, policymakers, and health advocates. By identifying the knowledge gaps, behaviors, and influencing factors related to coarse grain consumption, this study may provide valuable insights and suggestions for addressing the issue of low coarse grain consumption among Chinese citizens.

## Author contributions

The authors’ responsibilities were as follows – XL: conceptualization; JX, JL, GM, MW, YL, YH, KX: research; JX, MW, XL: data and statistical analyses; TT, NY, QW: participant recruitment; JX: writing original draft; JC, XL: writing review and editing. All authors have read and approved the final manuscript.

## Funding

This research was funded by the National Natural Science Foundation of China (82173504, 82011530197).

## Data availability

Data described in the manuscript, code book, and analytic code will be made available upon reasonable request pending approval.

## Conflict of interest

The authors report no conflicts of interest.
